# Poly(γ-glutamic acid)/Silica Hybrids with Calcium Incorporated in the Silica Network by Use of a Calcium Alkoxide Precursor

**DOI:** 10.1002/chem.201304013

**Published:** 2014-05-18

**Authors:** Gowsihan Poologasundarampillai, Bobo Yu, Olga Tsigkou, Daming Wang, Frederik Romer, Vineet Bhakhri, Finn Giuliani, Molly M Stevens, David S McPhail, Mark E Smith, John V Hanna, Julian R Jones

**Affiliations:** [a] Department of Materials, Imperial College LondonSouth Kensington, London, SW7 2AZ (UK) E-mail: gowshp@gmail.comjulian.r.jones@imperial.ac.uk; [b] Department of Bioengineering, Imperial College LondonSouth Kensington, London, SW7 2AZ (UK); [c] Department of Physics, University of WarwickCoventry, CV4 7Al (UK); [d] Lancaster University, Vice-Chancellor's OfficeLancaster, LA1 4YW (UK)

**Keywords:** bioactivity, calcium methoxyethoxide, hybrid materials, poly(γ-glutamic acid), sol-gel process

## Abstract

Current materials used for bone regeneration are usually bioactive ceramics or glasses. Although they bond to bone, they are brittle. There is a need for new materials that can combine bioactivity with toughness and controlled biodegradation. Sol-gel hybrids have the potential to do this through their nanoscale interpenetrating networks (IPN) of inorganic and organic components. Poly(γ-glutamic acid) (γ-PGA) was introduced into the sol-gel process to produce a hybrid of γ-PGA and bioactive silica. Calcium is an important element for bone regeneration but calcium sources that are used traditionally in the sol-gel process, such as Ca salts, do not allow Ca incorporation into the silicate network during low-temperature processing. The hypothesis for this study was that using calcium methoxyethoxide (CME) as the Ca source would allow Ca incorporation into the silicate component of the hybrid at room temperature. The produced hybrids would have improved mechanical properties and controlled degradation compared with hybrids of calcium chloride (CaCl_2_), in which the Ca is not incorporated into the silicate network. Class II hybrids, with covalent bonds between the inorganic and organic species, were synthesised by using organosilane. Calcium incorporation in both the organic and inorganic IPNs of the hybrid was improved when CME was used. This was clearly observed by using FTIR and solid-state NMR spectroscopy, which showed ionic cross-linking of γ-PGA by Ca and a lower degree of condensation of the Si species compared with the hybrids made with CaCl_2_ as the Ca source. The ionic cross-linking of γ-PGA by Ca resulted in excellent compressive strength and reduced elastic modulus as measured by compressive testing and nanoindentation, respectively. All hybrids showed bioactivity as hydroxyapatite (HA) was formed after immersion in simulated body fluid (SBF).

## Introduction

Bioactive glasses bond to bone, have been shown to stimulate osteogenesis and are available commercially in a particle form.[1] The ability to bond with bone is thought to be due to the formation of a hydroxycarbonate apatite (HCA) surface layer on reaction with body fluid.[2] Using the sol-gel foaming process, scaffolds have been developed that have an interconnected pore structure suitable for vascularised bone ingrowth with compressive strength in excess of 2 MPa.[3] However, as is the case for other porous bioceramics, the brittle nature of bioactive glasses limits their application to non-load-bearing bone defects. The synthesis of organic/inorganic hybrids is one strategy to mimic the natural nanocomposite structure of bone. The aim is to create porous scaffold materials that are bioactive, can share load with the bone and have tailored congruent degradation. Hybrids are synthesised by incorporating a polymer into the sol-gel process in an early (sol) stage of the process, as the silica network is being formed, so that the inorganic and organic species interact at the nanoscale. Several hybrids have been created by sol-gel routes in the past 15 to 20 years.[4]

Hybrids are classified as class I or II according to the interfacial bonding present between the organic and inorganic components. Class I hybrids only have weak van der Waals forces, hydrogen bonding and/or ionic bonding, whereas class II hybrids have strong covalent bonding,[4b] which is normally achieved by modifying the polymer so it can bond to the inorganic phase. Recently, organic/inorganic hybrids have been developed by using poly-γ-glutamic acid (γ-PGA) as the organic component.[4p, 5] A class II γ-PGA/silica hybrid was achieved by initially functionalising the γ-PGA with glycidoxypropyltrimethoxy silane (GPTMS) before the polymer was added to the silica sol. The epoxy ring of the GPTMS was hypothesised to open and react with the carboxylic acid group of the γ-PGA (nucleophilic attack),[6] leaving the γ-PGA functionalised with the methoxysilane groups, which hydrolyse and undergo condensation with the Si–OH groups in the tetraethylorthosilicate (TEOS)-based sol, linking the γ-PGA to the silica network by Si–O–Si bonds.

Inclusion of calcium in the hybrid system is important as it is vital to the formation of the hydroxycarbonated apatite (HCA) layer. Together with Si, calcium is also thought to act as a signalling agent to stimulate differentiation of osteogenic cells to bone cells as it is released from bioactive glass.[3a] However, traditional sol-gel calcium sources are not suitable for low-temperature hybrid materials. Calcium nitrate is the most popular calcium source for bioactive sol-gel glasses but a gel must be heated to over 400 °C to remove the nitrate byproducts.[7] As biodegradable polymers will be degraded at such high temperatures, an ideal calcium source would allow calcium incorporation at room temperature. Calcium chloride is another salt precursor of calcium that was previously used to synthesise organic/inorganic hybrids.[4p, 5b, 8] Yu et al. showed that when calcium salts were used, Ca was not incorporated into the silica network until a temperature of 400 °C was reached.[7b] Poologasundarampillai et al. also demonstrated that a moisture-assisted drying was required to allow Ca to enter the organic network (chelate) when using CaCl_2_ as the calcium source.[5b] One alternative route is to introduce the calcium in the polymer component,[5c] then introduce the calcium salt form of the polymer into the sol. The advantage of this method over using the free acid form of γ-PGA was that the calcium salt γ-PGA is soluble in the sol, so no other solvent was needed. Drawbacks were that the amount of calcium that could be incorporated was low and the calcium was associated with the carboxylic acid groups, which reduced the number of functional groups available for covalent coupling, and that Ca was not incorporated with the inorganic silica network. Therefore, a new calcium source is required that does not release toxic byproducts and can be incorporated into the hybrid system, both organic and inorganic, at low temperature (e.g., lower than 60 °C). One such calcium source is calcium methoxyethoxide (CME). Previously, we showed that for a sol-gel bioactive glass made with CME as the calcium source, the Ca was incorporated into the silica network at room temperature.[7b] The glass structure was studied as a function of heat-treatment temperature by using X-ray diffraction, which showed the diffraction patterns for the glasses to be identical at temperatures from ambient to 600 °C for the glasses with CME as the calcium source. Ion dissolution from the bioactive glasses also was observed not to change after heat treatment at different temperatures up to 600 °C, suggesting that low-temperature incorporation of calcium was successful when CME was the calcium source. Here, the aim is to investigate the use of CME as the calcium source for the synthesis of class II calcium silicate/γ-PGA hybrids. The properties of the hybrids synthesised with CME are compared with samples made with calcium chloride.

## Results and Discussion

Release of soluble Si and Ca ions from bioactive glass are beneficial to the regeneration of bone.[9] Scaffold materials for bone regeneration should be able to release these elements at a controlled rate. Previously, Poologasundarampillai et al. electrospun silicate/poly-l-lactic acid hybrid material fibre mats by using CME as the calcium source.[10] There, the CME was mixed with the electrospinning solution in the last seconds of the procedure to avoid the premature gelation that is due to the high reactivity of CME with water and hydroxyl groups. Here, the challenge is to produce γ-PGA/silica hybrids by using CME as the calcium source when the silica content is ∼50 wt % and so that the calcium is incorporated homogeneously within the organic and inorganic networks. Owing to the high reactivity of CME with water in the sol-gel process, its incorporation was not trivial; hence, this will be first explained in more detail.

### Determination of hybrid synthesis procedure

**Scheme 1:**
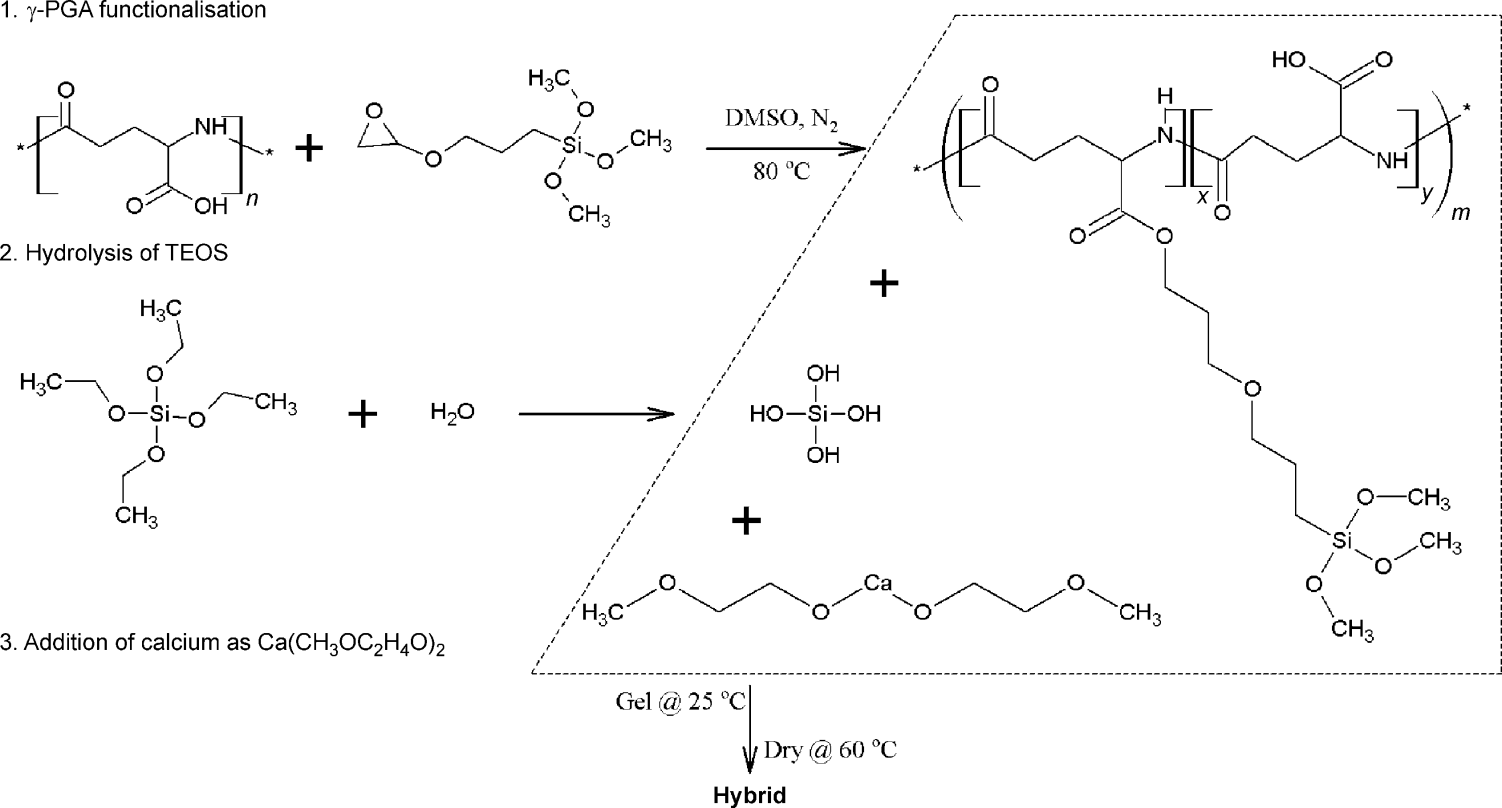
Schematic of the 3-pot synthesis of Class II γ-PGA/bioactive silica hybrids using calcium methoxyethyoxide (CME) as the calcium source.

Several systematic experiments were conducted to devise a viable method of incorporating CME into the γ-PGA/silica hybrid. Several important observations were made. CME was found to react with functionalised polymer solution (γ-PGA reacted with GPTMS) and also with hydrolysed TEOS to cause premature and heterogeneous gelation. This occurred immediately when CME was mixed with the functionalised polymer solution (γ-PGA+GPTMS) as calcium alkoxides have high reactivity with carboxylic acid groups,[11] which are present on γ-PGA. When a reduced *R* ratio (*R*=moles of H_2_O/moles of TEOS) of 2:1 was used for the hydrolysis of TEOS, gelation could be slowed to several minutes (<20 min) depending on the speed of addition of CME and the rate of mixing of the solution. Therefore, when making Class II hybrids with CME, as shown in Scheme 1, γ-PGA was first functionalised with GPTMS in DMSO, this was then added to hydrolysed TEOS to form the hybrid precursor solution. CME was then added slowly; however, this was also found to lead to rapid gelation, but by diluting CME with DMSO at a 1:1 volume ratio and by adding the diluted CME solution slowly to the hybrid precursor solution, the homogeneity of the reaction and gelation was improved. The gelation time for these hybrids was 5 min. In contrast, the hybrid systems made with CaCl_2_ as the calcium source gelled after 3 days (with HF also added). The faster gelation of the CME hybrid system was due to the rapid hydrolysis and condensation of CME. The use of HF in the CME hybrid system did not affect the gelation, an observation that might be due to HF being inhibited by the presence of CME. Magic-angle spinning (MAS) NMR spectrometry, X-ray diffraction, FTIR spectroscopy, secondary ion mass spectrometry (SIMS) and SEM were used to study the chemical, nano- and macro-structures of the hybrids produced with this synthesis route. The hybrids are denoted as 2ECCa_ME_, where 2 corresponds to two moles of glutamic acid (E) to GPTMS (C), with calcium (Ca) from either CME (ME) or CaCl_2_ (Cl). Samples made without the addition of HF are denoted as 2ECCa_ME_*. Calcium-free hybrids are denoted as 2EC and were produced following the same procedure as that for the synthesis of 2ECCa_ME_, but no calcium was added.

### Atomic-scale structure

**Figure 1:**
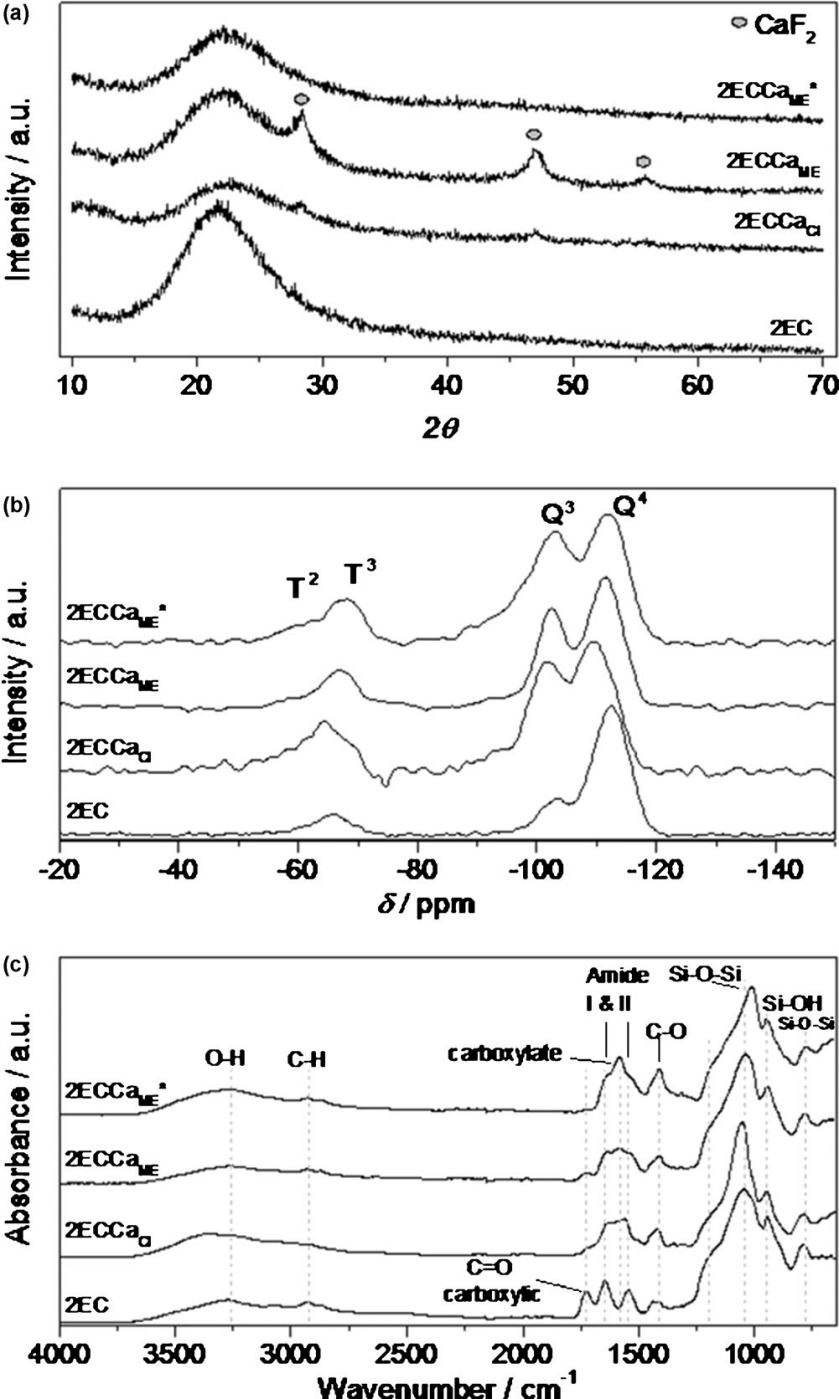
Characterisation of Class II γ-PGA/bioactive silica hybrids synthesised with calcium chloride (2ECCa_Cl_) or calcium methoxyethoxide (2ECCa_ME_) or without calcium (2EC) with molar ratios of γ-PGA/GPTMS of 2: (a) XRD (b) ^29^Si MAS NMR spectra and (c) FTIR spectra. Samples where HF was not added are marked with *.

XRD patterns (Figure 1 a) show that 2EC and 2ECCa_ME_* were amorphous but 2ECCa_Cl_ and 2ECCa_ME_ contained some low-intensity crystalline peaks that were identified as CaF_2_. This indicates that addition of HF to the calcium-containing samples led to the formation of CaF_2_ crystals. This was previously shown to occur in 2ECCa_Cl_ by XRD and TEM.[5b] It seems that CaF_2_ also forms in the case of the 2ECCa_ME_ sample, but the peaks were broader and much more intense, indicating that there were larger numbers of smaller CaF_2_ crystals present in the 2ECCa_ME_ sample compared with 2ECCa_Cl_. The formation of CaF_2_ by the reaction between HF and CME explains why HF does not accelerate gelling when it is added to the hybrid precursor solution as the fluoride is consumed to form CaF_2_.

### Silica-bonding structure

Figure 1 b shows the ^29^Si MAS NMR spectra for the four samples. All the samples showed the presence of both T^*n*^ and Q^*n*^ species (T^*n*^ and Q^*n*^ correspond to the structures of CSi(OSi)_*n*_(R)_3−*n*_ and Si(OSi)_*n*_(R)_4−*n*_, respectively, where R is a non-bridging oxygen).[12] The results of the Gaussian fitting of T^*n*^ and Q^*n*^ distributions to the data of all the ^29^Si MAS NMR spectra are summarised in Table 1. The ratio of T^3^/T^tot^ and Q^4^/Q^tot^ in Table 1 show the degree of condensation of the GPTMS and silica network respectively, and *D*_c_ represents the degree of condensation of all Si–O species in the hybrid. Large variation between the two ratios and *D*_c_ for the different samples suggests that the hybrids had different atomic-scale structures, a fact that could reveal information about the reaction and gelation mechanism. The 2ECCa_ME_ and 2EC samples had a high T^3^/T^tot^ ratio (>0.9) compared with the 2ECCa_Cl_ and 2ECCa_ME_* samples (0.6), indicating that the GPTMS in 2ECCa_ME_ and 2EC underwent condensation with hydrolysed TEOS or with itself to a greater extent. For the TEOS-derived silica network, the Q^4^/Q^tot^ ratio was high for 2EC (0.8), the ratio was lower for 2ECCa_Cl_ and 2ECCa_ME_ (0.6) and finally 2ECCa_ME_* had the lowest value of 0.4. The 2EC sample has the highest T^3^/T^tot^, Q^4^/Q^tot^ and *D*_c_ (95 %) values, which suggests that the silica species in 2EC were highly condensed. The calcium-containing, HF-free CME hybrid, 2ECCa_ME_*, had the lowest T^3^/T^tot^, Q^4^/Q^tot^ and *D*_c_ (83 %) values of all the samples, indicating that a polymeric chain-like silica structure or a network composed of primary silica particles may have been formed. In the case of the 2EC sample, a rather coarse particle-like sol-gel structure formed. The rapid reaction of CME with water means that the 2ECCa_ME_* and 2ECCa_ME_ samples gelled rapidly in comparison to 2EC and 2ECCa_Cl_, therefore the CME-derived samples had a highly disrupted, low condensed silica network, an observation that is in agreement with the ^29^Si NMR data. However, the 2ECCa_ME_ sample had a higher degree of condensation (*D*_c_=90 %) than expected. This could be due to the addition of HF prior to the addition of CME to the hybrid precursor sol. HF was added to the hydrolysed TEOS and functionalised γ-PGA mixture and allowed to react for 3 min. Here, HF catalyses the condensation of hydrolysed TEOS and GPTMS, forming silica particles with a higher proportion of Q^4^ species,[13] but with the addition of CME the precursor solution gels, trapping HF and all constituents in the gel. However, another reason for the increased *D*_c_ of 2ECCa_ME_ may be due to the formation of CaF_2_. Calcium from CME could enter the silicate network if HF was not added, but instead reacted with F^−^ to form CaF_2_, a process that reduced the number of network modifiers in the silicate network, increasing its connectivity. Therefore, it has the second highest degree of condensation, that is, second only to 2EC, which has no Ca.

**Table 1:** Chemical shifts and relative proportions of T^*n*^ and Q^*n*^ species in Class II γ-PGA/bioactive silica hybrids synthesised with calcium chloride (2ECCa_Cl_) or calcium methoxyethoxide (2ECCa_ME_) or without calcium (2EC) with processing temperatures of 60 °C. Samples where HF was not added are marked with ^*^. The errors in the chemical shift (*δ*) is ±0.5 ppm, in the intensity is ±1 %, in the ratios is ±0.05 and in *D*_c_ is ±3 %

Samples	T^2^		T^3^		Q^2^		Q^3^		Q^4^		T^3^/T^tot^	Q^4^/Q^tot^	*D*_c_
	*δ*	*I*	*δ*	*I*	*δ*	*I*	*δ*	*I*	*δ*	*I*			
	[ppm]	[%]	[ppm]	[%]	[ppm]	[%]	[ppm]	[%]	[ppm]	[%]			
2EC	–	0.0	−65.4	11.7	–	0.0	−103.0	18.1	−112.4	70.2	1.0	0.8	95
2ECCa_Cl_	−58.0	8.0	−65.1	13.0	−93.1	5.2	−101.3	29.2	−109.8	44.6	0.6	0.6	87
2ECCa_ME_	−59.0	2.0	−66.9	13.9	−94.4	3.8	−102.5	30.5	−111.4	49.7	0.9	0.6	90
2ECCa_ME_^*^	−61.4	6.9	−68.2	9.9	−95.0	13.4	−103.0	33.2	−112.2	36.5	0.6	0.4	83

Hybrid sample 2ECCa_Cl_, which was produced with calcium chloride as the calcium source, had a similar *D*_c_ to that of 2ECCa_ME_, although not as much CaF_2_ is formed as in 2ECCa_ME_. This suggests that the Ca from CaCl_2_ stayed in the precursor solution and it had not entered the silica network. This is in agreement with observations by Yu et al.[7b]

### Chemical structure

Figure 1 c shows the FTIR spectra of the hybrids. The bands at 1045 and 790 cm^−1^, corresponding to Si–O–Si, and that at 947 cm^−1^, corresponding to Si–O from the inorganic component of the hybrids, were observed in all spectra. In addition, no bands corresponding to the oxirane ring were observed, indicating that ring opening was successful in all the hybrids. All the samples exhibited the band corresponding to amide I and II from γ-PGA, whereas the band corresponding to the free carboxylic acid at 1725 cm^−1^ (C=O) was only visible in the calcium-free 2EC sample. However, in the calcium-containing samples, the band corresponding to the carboxylate anion (–COO^−^) at 1580 cm^−1^ was present. This indicates that the calcium cross-linked the γ-PGA at the carboxylic acid groups, forming a Ca-γ-PGA salt. The ionic cross-linking of the γ-PGA by Ca^2+^ could be beneficial in the hybrid as it could lead to stronger hybrid materials.[5c] Qualitatively, the FTIR spectra also show that a higher proportion of the carboxylic acids in γ-PGA were converted to carboxylate anions in the HF-free 2ECCa_ME_* hybrid compared with 2ECCa_Cl_ and HF-containing 2ECCa_ME_. This could be due to the HF added to the 2ECCa_Cl_ and 2ECCa_ME_ instead using Ca^2+^ ions to form CaF_2_ as shown by XRD, that is, the F^−^ and COOH groups compete for the Ca^2+^ during the hybridisation reactions. 2ECCa_ME_* was therefore selected for SIMS, SEM, compression mechanical testing and cell culture testing.

### Compositional imaging

**Figure 2:**
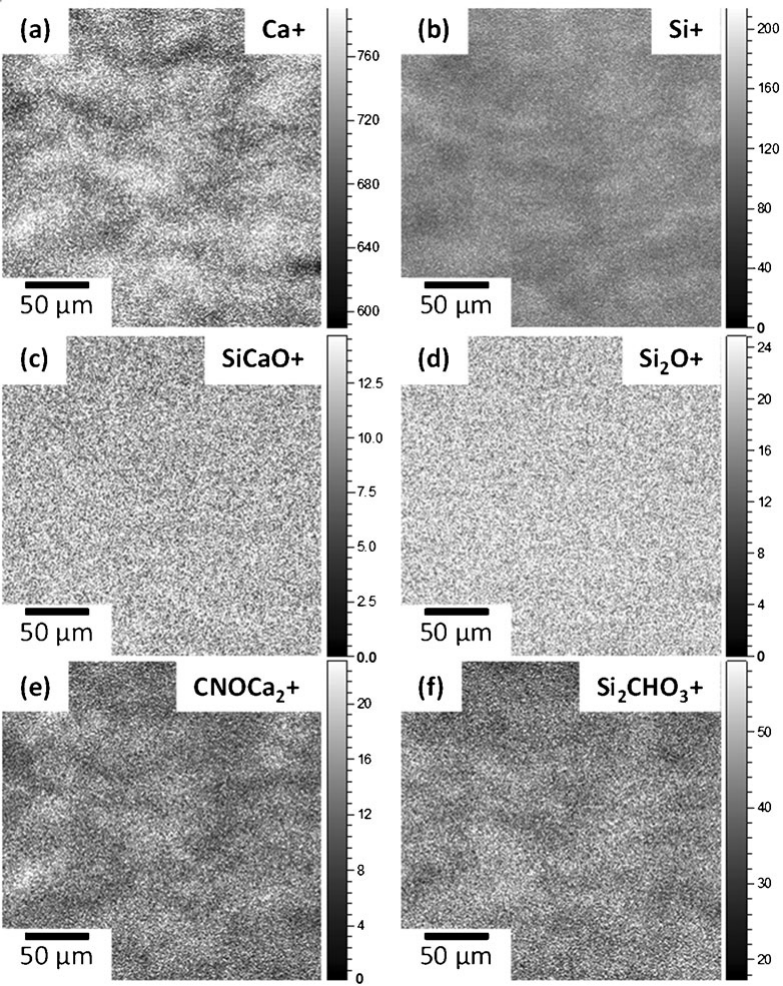
TOF SIMS images of 2ECCa_ME_*: (a) Ca^+^, (b) Si^+^, (c) SiCaO^+^, (d) Si_2_O^+^, (e) CNOCa_2_^+^ and (f) Si_2_CHO_3_^+^.

Figure 2 shows the time-of-flight (TOF) SIMS surface images of the 2ECCa_ME_* hybrid. Figure 2 a and b show the Ca^+^ and Si^+^ mapping, respectively. Total count for Ca^+^ was 4.8×10^7^, which was higher than that of Si^+^ (9.0×10^6^); this was due to the Ca^+^ being easily ionised compared to Si^+^. The elemental distribution images show that at the macroscale the Ca and Si were not homogeneous in 2ECCa_ME_*. The Ca count was found to be low in regions where Si was high. However, Figure 2 c and d, of Si–O–Ca^+^ and O–Si–O^+^ molecules from the inorganic silica network show that the Ca was homogeneously distributed in the silica network, which was also homogeneous. This suggests that the CME reacts with the hydrolysed TEOS and enters the Si network at room temperature as observed in silica gels by Yu et al.[7b] A continuous Si network was formed in the hybrid and Ca was homogeneously distributed within this network. Finally, Figure 2 e and f show the molecular distribution profile of CNCa_2_O^+^ from calcium bonded to the γ-PGA and that of CSi_2_O_3_H^+^ from condensed GPTMS, respectively. These show identical profiles to that of Ca^+^ and Si^+^. Therefore, the inhomogeneity seen in Figure 2 a and b was due to the phase separation of Ca-cross-linked polymer and condensed GPTMS molecules.

To summarise, during functionalisation of γ-PGA, the epoxy ring of the GPTMS is expected to react with –COOH groups of the γ-PGA, forming covalent bonds between GPTMS and γ-PGA, whereas for some GTPMS molecules, the epoxide ring is expected to open without nucleophilic attack, forming a diol, and a proportion of GPTMS may condense with itself, forming dimers or trimers.[6] Once hydrolysed TEOS is added, the Si–OH groups on hydrolysed GPTMS molecules are expected to complete coupling to the silica network through polycondensation, but additional silica condensation on the GPTMS also took place. On addition of CME, the CME reacted with all the remaining free –COOH sites of the γ-PGA, which then cross-linked with Ca. At the same time, CME also condensed with the hydrolysed TEOS and gelation occurred, locking in place the self-condensed GPTMS and Ca-cross-linked γ-PGA. This leads to a structure where the Si network derived from TEOS is homogeneous, Ca that has reacted with and entered this inorganic network is also homogeneous, whereas the GPTMS that has reacted with itself and the free carboxylic acid groups of γ-PGA, which is cross-linked by Ca, are not homogeneous in the hybrid.

### Morphology of hybrids

**Figure 3:**
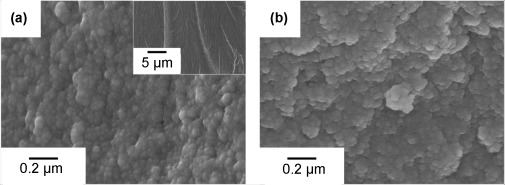
SEM images of 2ECCa_ME_* before (a and inset) and after (b) soaking in TRIS buffer solution.

Figure 3 shows SEM images of 2ECCa_ME_* before (Figure 3 a) and after soaking in tris(hydroxymethyl)aminomethane (TRIS) buffer solution (Figure 3 b). The as-made hybrid fracture surface had a smooth surface topography (inset of Figure 3 a) and high-magnification images showed that it is composed of the typical sol-gel silica morphology of bonded silica nanoparticles[7a] but with polymer packed in between the particles. The particles were estimated from the image to be <50 nm in size. Similar nanostructures were found for the 2ECCa_Cl_ and 2ECCa_ME_ hybrids (not shown).

### Mechanical testing of hybrid monoliths

**Figure 4:**
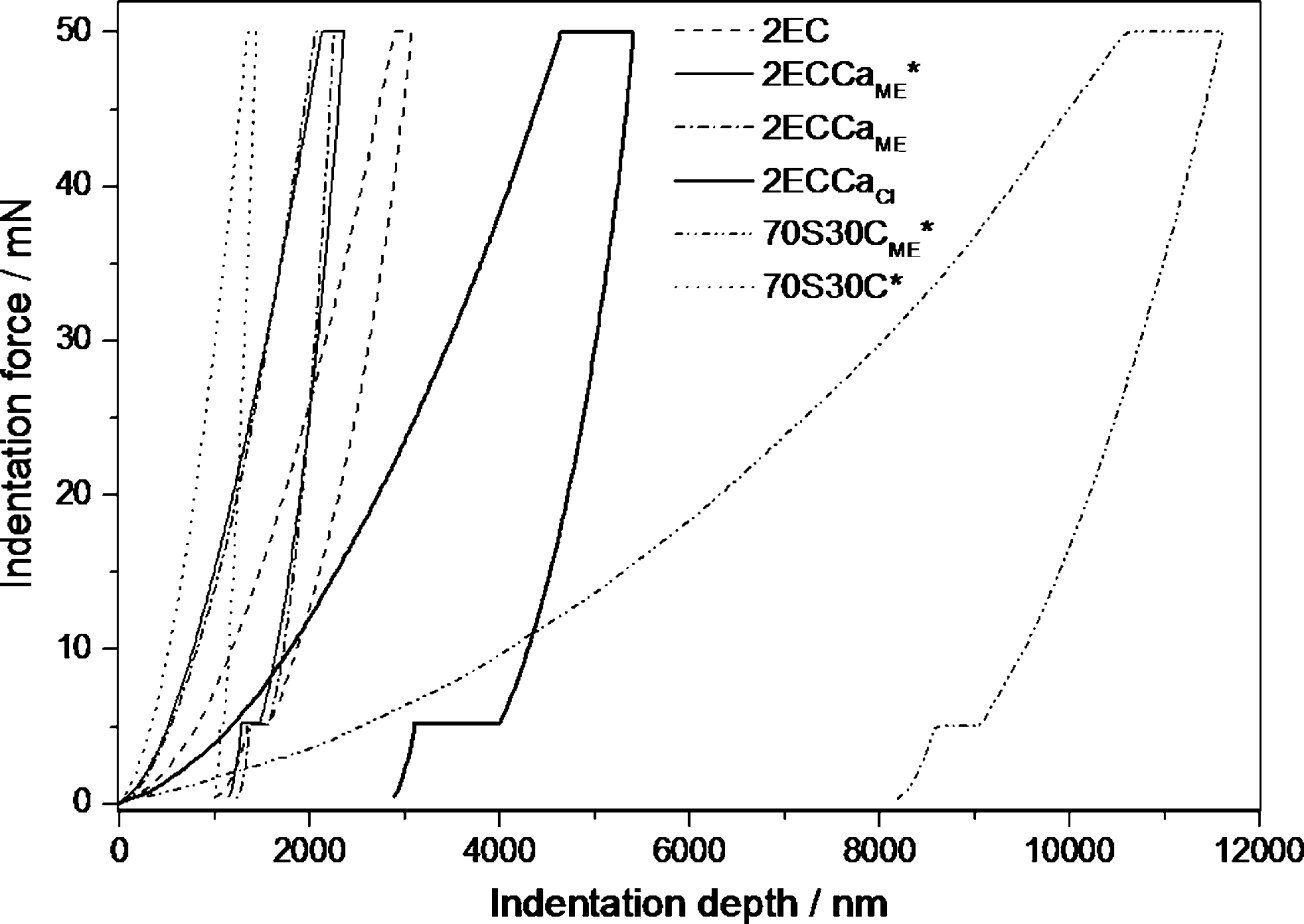
Loading and unloading nanoindentation curves of the hybrids synthesised with calcium methoxyethoxide (2ECCa_ME_), calcium chloride (2ECCa_Cl_) and without calcium (2EC). Traditional 70S30C bioactive glass synthesised with calcium nitrate sintered at 700 °C and 70S30C_ME_* processed at 60 °C are included for comparison.

Figure 4 shows the typical nanoindentation loading and unloading curves obtained for the hybrids and 100 wt % inorganic glass samples (70S30C_ME_*: 70 mol % SiO_2_, 30 mol % CaO). The testing procedure adopted in this study resulted in crack-free deformation of the hybrid and 70S30C_ME_* samples, an observation that was proven by the typical force versus depth curve observed with a Berkovich indenter (Figure 4). The organic content in the hybrid material could give rise to viscoelastic effects that would be observed on the unloading curve as a “nose”;[14] this was not observed in the case of these hybrids under the testing conditions used. The dwell at 50 mN for 20 s allowed for complete creep relaxation, hence a “nose” was not observed.

The reduced elastic modulus (Er) and hardness values from the force–depth curves[15] and the creep parameter *A*/*d*(0)[14] calculated from the dwell data at 50 mN are summarised in Table 2. The reduced modulus of the 70S30C glass was at least 2.5 times greater than that of the hybrids. This was expected as the 70S30C was heated to 700 °C, which leads to a highly condensed silicate structure, whereas the hybrids were only heated to 60 °C. However, all the hybrid samples have much higher modulus (×8) than that of the 100 wt % inorganic, 70S30C_ME_*, sample synthesised with CME as the calcium source and processed at 60 °C. This shows that the hybridisation with an organic polymer was very effective at increasing the mechanical properties of this inorganic system.

**Table 2:** Organic content, reduced modulus, hardness and creep parameter *A*/*d*(0) of 70S30C made with Ca(NO_3_)_2_ (70S30C) and with calcium methoxyethoxide (70S30C_ME_) and Class II γ-PGA/bioactive silica hybrids synthesised with calcium methoxyethoxide (2ECCa_ME_), calcium chloride (2ECCa_Cl_) and without (2EC) with a processing temperature of 60 °C. Samples where HF was not added are marked with ^*^

Sample	Organic [wt %]	Reduced modulus [GPa]	Hardness [GPa]	Creep parameter *A*/*d*(0)
2EC	47	4.04±0.24	0.45±0.04	0.020±0.001
2ECCa_ME_^*^	47	8.99±0.84	0.64±0.04	0.035±0.002
2ECCa_ME_	47	7.99±2.01	0.52±0.06	0.040±0.006
2ECCa_Cl_	50	3.51±1.19	0.16±0.04	0.071±0.013
70S30C_ME_^*^	0	0.47±0.07	0.02±0.01	0.034±0.005
70S30C	0	24.40±0.60	1.39±0.06	0.018±0.001

The reduced modulus values of 2EC and 2ECCa_ME_*, 2ECCa_ME_ and 2ECCa_Cl_ were calculated to be 4.04, 8.99, 7.99 and 3.51 GPa, respectively. The addition of Ca in the hybrids using CME as the source more than doubled the modulus, whereas CaCl_2_ reduced it. This suggests that CME as the calcium source was effective in incorporation of Ca in the hybrid. During the synthesis of the 2ECCa_ME_ hybrids, as soon as CME was added it reacted with the inorganic silica network and the free carboxylic acid groups of γ-PGA. This ionic cross-linking by Ca^2+^ of the polymer was also observed when CaCl_2_ was used as the calcium source, hence it seems that the improved mechanical properties of the 2ECCa_ME_ hybrids could be due to Ca cross-linking both the organic and the inorganic components separately as well as the to each other, thereby forming a true interpenetrating network of organic and inorganic constituents. This conclusion is fair as the 2ECCa_ME_* has a higher Er than the organic-free 70S30C_ME_* and the Ca-free 2EC samples. Mammeri et al. performed nanoindentation studies on class II poly(methyl methacrylate) (PMMA) /SiO_2_ hybrids and found a modulus of 6.60 GPa and hardness of 0.54 GPa.[16] The class II γ-PGA/SiO_2_ hybrids, 2EC, synthesised in this study had a lower relative modulus, perhaps owing to the inherent stiffness of the PMMA and from the higher curing temperature of 100 °C used in the Mammeri study. However, the calcium-containing 2ECCa_ME_* had a higher modulus compared with PMMA/SiO_2_. This suggests that careful choice of the organic component and how it is cross-linked in the hybrid have a great influence on the overall mechanical properties.

Table 2 also shows that the relative modulus of 2ECCa_ME_ was similar to 2ECCa_ME_*. This indicates that the addition of HF did not influence the mechanical properties of the hybrids, an observation that supports the finding that addition of HF did not change the gelation behaviour of the hybrid.

Beake[17] showed that by fitting the logarithmic equation


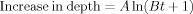
(1)

to the 20 s dwell data, at the maximum load of 50 mN, the extent and rate of inelastic deformation can be obtained, where *t* is the dwell time, *A* is the extent term and *B* is the rate term. Beake showed that the normalised *A*/*d*(0) term, where *d*(0) is the initial deformation, can be used to compare the extent of inelastic deformation of different materials. The *A*/*d*(0) values for the hybrids and 70S30C samples are given in Table 2. Sample 2EC and 70S30C heated to 700 °C have the smallest *A*/*d*(0) values, suggesting that there was no significant creep in the 2EC sample compared with other samples included in this work. The *A*/*d*(0) value for the 2ECCa_ME_ and 2ECCa_ME_* hybrids were 0.035 and 0.040, respectively, whereas for the 2ECCa_Cl_ hybrid it was 0.071. This suggests that the CaCl_2_-derived hybrid was much more viscoelastic compared with the CME-derived hybrids. Therefore the structure of 2ECCa_Cl_ could be one where the organic phase has more freedom of movement than that in the 2ECCa_ME_ samples. Hence, in the 2ECCa_ME_ hybrids, the organic component was much more cross-linked than in 2ECCa_Cl_. As previously discussed, the increased cross-linking could possibly be through the Ca^2+^ to γ-PGA and/or to silica.

Compression tests were performed on cylindrical hybrid samples of 2ECCa_ME_*. Figure S1 in the Supporting Information shows the stress–strain curves of 2ECCa_ME_* samples with three different height/width ratios (H/W), as well as those for 70S30C stabilised to 800 °C and 2ECCa_Cl_. 70S30C was included as previous work has shown bioactive glass scaffolds (70 mol % SiO_2_, 30 mol % CaO) to have optimal compressive strength when the glass was sintered to 800 °C.[3b] Therefore, monoliths of this glass and a 2ECCa_Cl_ sample were used as a comparison. When the height-to-width ratios were 1:2 and 1:1, compression tests showed that the 2ECCa_ME_* samples had higher strength and elastic modulus compared with the 2ECCa_Cl_ hybrid and that they had comparable strength to 70S30C even though the hybrids had ∼50 wt % inorganic material. Hybrid sample 2ECCa_ME_* with a H/W ratio of 2:1 failed at a lower strength than the other 2ECCa_ME_* samples, owing to the large height-to-width ratio, which made it fail at lower loads from higher inherent defects within. Hybrid 2ECCa_ME_* with a ratio of 1:2 had the highest strain-to-failure value (>70 %) owing to the sample being wider than it was high. This shows that the compressive test is very sensitive to the sample dimensions and that it could lead to false interpretations of strength in hybrid materials. Figure S1 b in the Supporting Information shows the yield stress as a function of H/W of the 2ECCa_ME_* hybrids. This clearly shows that the yield stress decreased with increasing H/W, hence samples with smaller height compared with width (2ECCa_ME_*, H/W of 1:2) show higher strength and strain. The hybrid samples failed by cracking along the direction of the applied force. Once a crack formed, it travelled through the samples in the direction parallel to the force, resulting in a chipped sample. The effective cross-sectional area of the remaining sample was then reduced and hence the samples failed at lower loads before reaching its maximum failure load. Therefore, a sample with a high H/W ratio would fail at lower loads and a sample with a low H/W ratio would withstand the loading for longer before complete failure. Therefore, care should be taken when interpreting the results of the compressive test data of these hybrid monoliths. However, in the case of a porous scaffold for bone regeneration, for which this hybrid material is being developed, compressive testing is important and should be performed. Following the international standard ISO 640:2003, the recommended test specimen dimensions of H/W>1 gave an elastic modulus of 2.0±0.3 GPa and compressive strength of 64.2±20 MPa for the 2ECCa_ME_* sample. If we assume that the mechanical properties of the produced porous scaffolds follow the cellular solids theory,[18] a 70 % porous scaffold is predicted to have a strength of 3.17 MPa. This is in the range of the strength of human trabecular bone, which has 2–12 MPa[19] compressive strength.

### Hybrid degradation and polymer release

**Figure 5:**
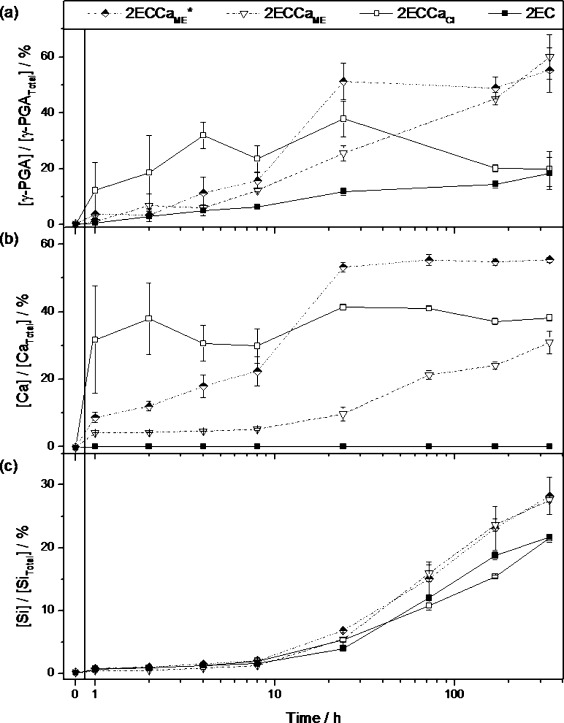
Release profile curves of (a) polymer, (b) Ca^2+^ ions and (c) soluble Si species from hybrids soaked in TRIS buffer solution for 2 weeks.

Soaking hybrids in TRIS buffer and analysing the polymer and ions released can reveal details about the structure and bonding present within the hybrid organic and inorganic constituents. Figure 5 shows the polymer release profile and Si species and Ca ion dissolution profiles of the class II hybrid samples soaked in TRIS buffer for 2 weeks. The concentration of the species released at a particular point in time was weighted against the total nominal concentration of that particular species in the sample. For example, [γ-PGA]/[γ-PGA_Total_] (%) means the concentration of γ-PGA in TRIS buffer solution at that point in time divided by the total concentration of γ-PGA in 150 mg of hybrid sample. Therefore, Figure 5 a shows the percentage of polymer released from the sample as a function of soaking time. Figure 5 b and c show the percentage of Ca and Si released from the sample as a function of soaking time, respectively. The calcium-free 2EC hybrid has a steady and slow release of γ-PGA. After 2 weeks of soaking, up to 20 % of γ-PGA had been released into the solution. The polymer release profile of the 2ECCa_Cl_ hybrid was more complex; it shows a burst of release of γ-PGA (>12 %) in the first hour of soaking, increasing to 40 % at 24 h. Then, a large reduction in concentration (18 %) was observed at 1 week. The initial burst of release indicates that a large portion of γ-PGA was loosely bound in the 2ECCa_Cl_ hybrid. The reduction in γ-PGA concentration in the TRIS solution after 24 h could be a result of γ-PGA cross-linking to itself (ionic and/or through GPTMS), leading to insolubility and precipitation. The calcium ion release profile of the 2ECCa_Cl_ hybrid is very similar to its polymer release profile. The majority of the calcium ion release took place in the first 2 h of soaking, accounting for the total ion released from the hybrid in the 2 weeks of soaking. This shows that the Ca^2+^ ion was also loosely bound in the hybrid and could be closely associated with the polymer.

The 2ECCa_ME_ hybrids with and without HF addition showed similar γ-PGA release profiles, where a steady increase in γ-PGA release was observed. The 2ECCa_ME_* sample deviated from this at 24 h soaking time, when a large amount of γ-PGA was released into solution from the 2ECCa_ME_* hybrid. This could be due to a physical breakup of the sample in solution; however, a corresponding Si release was not observed at this point in time. After 2 weeks of soaking, around 60 % of γ-PGA had been released from the 2ECCa_ME_ hybrid samples. The 2ECCa_ME_* sample showed a steady release of Ca^2+^ ions in the first 8 h of soaking, after which a rapid increase in Ca^2+^ concentration from 22 % to 53 % was observed, followed by a plateau in Ca^2+^ concentration up to 2 weeks. The profiles of both the release of γ-PGA and the dissolution of Ca^2+^ ions from the 2ECCa_ME_* hybrid were similar, indicating the calcium released into TRIS buffer was associated with γ-PGA. 2ECCa_ME_ showed a very slow calcium release for the first 24 h of soaking, during which less than 10 % of calcium was released. After 2 weeks, around 30 % of Ca^2+^ ions had been released. This was just under half of that released from the HF-free 2ECCa_ME_*. The difference in Ca^2+^ release between the CME samples was due to the addition of HF, which caused formation of CaF_2_. CaF_2_ is sparingly soluble in TRIS, therefore as a result of this Ca^2+^ was locked in the hybrid.

Finally, the release of soluble Si species from the hybrid is shown in Figure 5 c. Up until 8 h of soaking, very small amounts of Si species were released into the TRIS buffer solution from all of the samples. Following this, each of the samples showed a steady increase to more than 20 % Si release after soaking for up to 336 h. Both CME-derived samples, 2ECCa_ME_ and 2ECCa_ME_*, had identical Si release for the whole duration of testing.

To summarise, the dissolution study in TRIS buffer solution suggests that the 2ECCa_Cl_ samples were poorly hybridised as a burst of release of γ-PGA and Ca and slow release of Si were observed in the first few hours of soaking. This indicates that the Si network was strongly bonded whereas the organic network was loosely bonded in the hybrid. In the case of the CME-derived hybrids, steady release of all constituents was observed with instances of uncontrolled release behaviour. It also appears that higher proportions of Ca from CME were bound to γ-PGA owing to the reactivity of calcium alkoxide to carboxylic acid groups. Release of ∼60 % of γ-PGA from the CME samples within 336 h of soaking indicates that the γ-PGA was poorly cross-linked to the silica network. Better cross-linking of γ-PGA to silica is required before this material could be used in regeneration applications requiring scaffold support for the long-term (typically more than 6 months).

**Figure 6:**
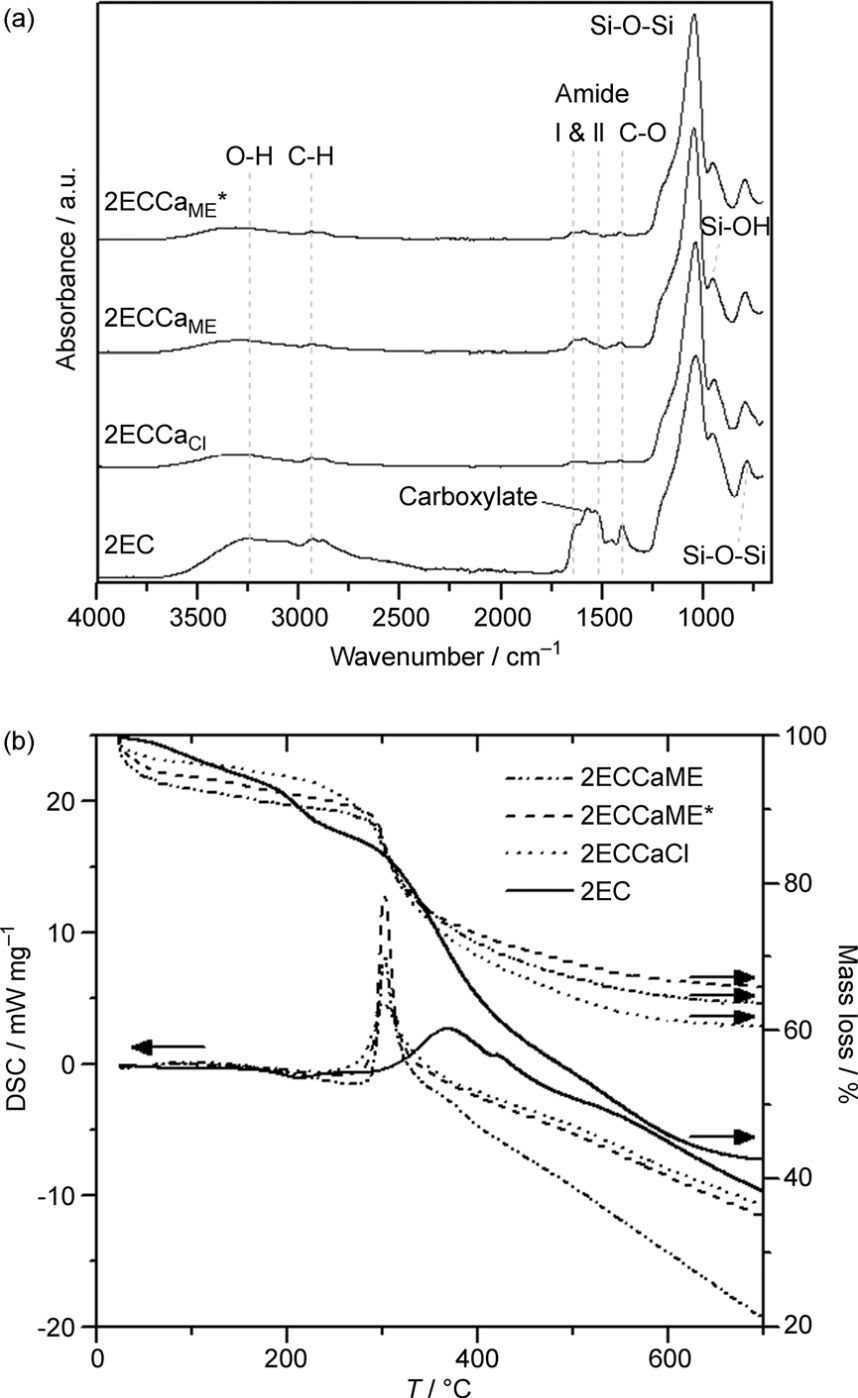
(a) FTIR spectroscopy and (b) TGA-DSC of the hybrids after 4 weeks in TRIS buffer solution.

Figure 6 shows FTIR spectra and thermogravimetric analysis–differential scanning calorimetry (TGA-DSC) plots for the samples after soaking in TRIS buffer for 4 weeks. All the FTIR spectra had clear bands corresponding to the Si–O–Si and Si–OH vibrations. The amide and C–O vibrations from the polymer were also clearly visible in the spectrum of the calcium-free 2EC sample. The calcium-containing hybrids have very weak bands at the amide, OH and CH vibrations, indicating the presence of some organic material. The TGA-DSC curve (Figure 6 b) shows that the organic content (γ-PGA+organic of GPTMS) of the 2EC sample was >50 wt % after soaking in TRIS buffer for 4 weeks. This is a slight increase in total organic content of the hybrid from the initial 46 wt % before soaking, a fact that means that higher proportions of inorganic (silica) than organic material had been released from the hybrid into the solution. This is supported by the Si and polymer dissolution rates in Figure 5. The calcium-containing samples 2ECCa_Cl_, 2ECCa_ME_ and 2ECCa_ME_* have similar inorganic content (about 70 wt %) after soaking in TRIS buffer for 4 weeks. Before soaking, the calcium-containing samples had just over 50 wt % inorganic content, this means that after soaking, a larger proportion of organic material was released. The opposite was observed for the 2EC sample, this indicates that the presence of Ca increased the dissolution of organic species in the hybrid. This could be due to the formation of calcium-polyglutamate, which is more soluble in aqueous media than the free acid γ-PGA.[20]

### Bioactivity

**Figure 7:**
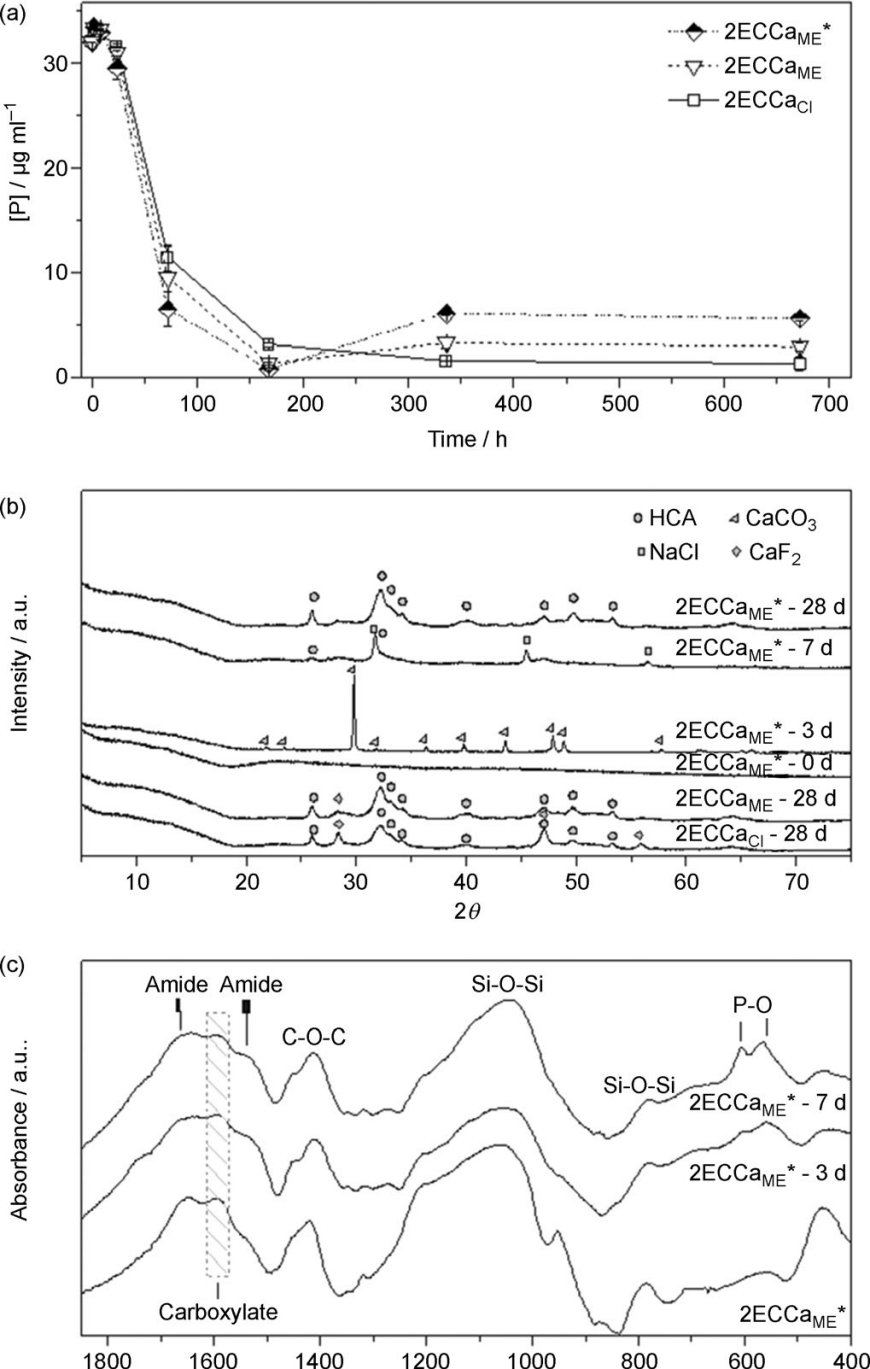
(a) Concentration of P in SBF with soaking time, (b) XRD and (c) FTIR spectra of the hybrids after 4 weeks in SBF solution.

Figure 7 a shows the concentration of phosphorus in simulated body fluid (SBF) after soaking the calcium-containing hybrid samples 2ECCa_ME_*, 2ECCa_ME_ and 2ECCa_Cl_. All three samples showed a rapid decrease in P concentration from 1 day to 3 days and a further drop from 3 days to 7 days. This indicates the deposition of phosphorus on the samples, which could correspond to the formation of a calcium phosphate layer on the sample. Figure 7 b shows the XRD patterns of the hybrids before and after soaking in SBF for 3, 7 and 28 days. Mature crystalline hydroxyapatite (HA) was found on all the calcium-containing 2ECCa hybrids. Diffraction patterns of the 2ECCa_ME_* (HF-free) hybrid before soaking and after soaking for 3, 7 and 28 days are also shown in Figure 7 b. It shows that after 3 days of soaking, CaCO_3_ was formed on the hybrid in SBF, a product that disappeared by 7 days as HA began to form. HA detection was supported by the FTIR curves (Figure 7 c), which show the hybrid sample 2ECCa_ME_* before and after soaking for 3 and 7 days in SBF. Bands corresponding to P–O vibrations (Figure 7 c) were observed after 3 days, bands that become intense and sharp after 7 days of soaking. This suggests a calcium orthophosphate phase was deposited on the sample by 3 days, the phase then matured to HA by 7 days (XRD) and grew into a more crystalline material by 28 days. FTIR also shows evidence of amide I and II and C–O–C vibrations up to 7 days, an observation that indicates that majority of the polymer was still present in the hybrid.

### Cell study

**Figure 8:**
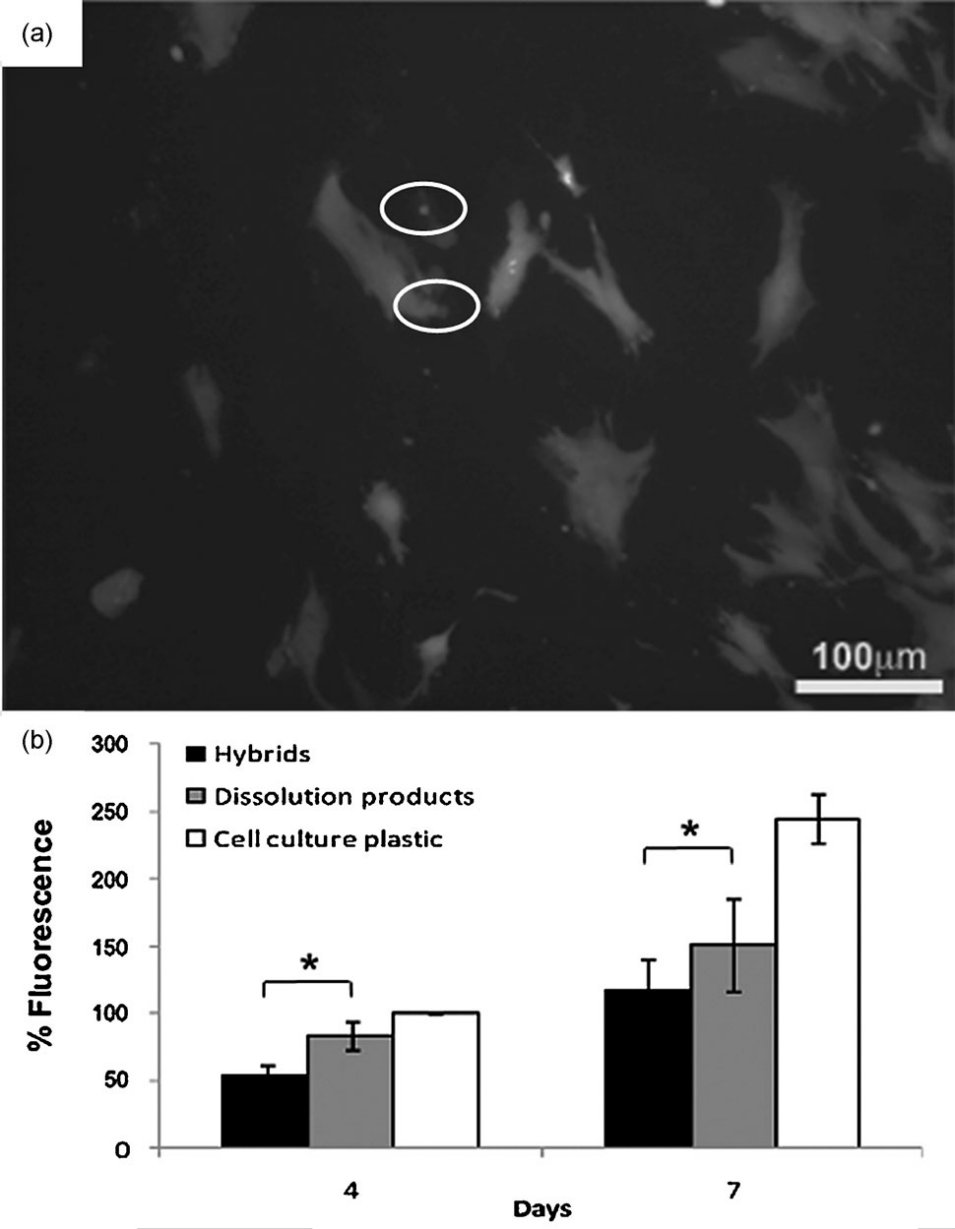
Human bone marrow derived mesenchymal stem cells (hMSCs) viability on 2ECCaME*. (a) LIVE/DEAD™ assay of the hMSCs cultured on the hybrids’ surface for seven days (dead cells circled). (b) Metabolic activity as measured with the Alamar Blue assay after four and seven days in culture. *=*p*<0.001.

To determine whether the hybrid samples were cytotoxic, human bone marrow derived mesenchymal stem cells (hMSCs) were cultured on 2ECCa_ME_ and the viability was examined with the LIVE/DEAD assay after 3 days (Figure 8 a). The assay revealed that there were numerous live hMSCs on the hybrids and very few dead cells. Additionally, from the LIVE/DEAD assay it was observed that the cells were well spread on the hybrids after 3 days in culture, indicating a favourable cell attachment. To further examine the effect of the hybrids on cell viability and growth, the Alamar Blue assay was used to measure the cell metabolic activity after 4 and 7 days. The metabolic activity of the cell monolayer on the tissue culture plastic around the hybrid samples was also measured to check if release from the samples resulted in cell death or number reduction (Figure 8 b). The Alamar Blue assay confirmed that the hybrids were not toxic to the hMSCs and further demonstrated that the cells grew on the hybrids over the period of 7 days, indicating a favourable cell response. In addition, the dissolution products of the hybrids were not toxic and also allowed the hMSCs to grow over time, indicating that there was no hindering of cell growth (Figure 8 b).

## Conclusion

Hybrid materials were successfully produced by using calcium methoxyethoxide (CME) as the Ca precursor. The CME was found to induce gelation on mixing with the organic/inorganic mixture. Once controlled, this was beneficial as rapid gelation is usually achieved by using HF as a catalyst, which is a hazardous reagent. Very little difference was found in terms of gelation time between the class II CME hybrids whether using HF or not. ^29^Si MAS NMR showed the presence of both T and Q species in the hybrids. It also showed that the Si–O–Si network connectivity was lower in the CME samples when HF was not used owing to Ca acting as a network modifier when introduced as CME. However, when HF was used, the Si–O–Si network connectivity increased. This was due to the combined influence of HF catalysing the condensation of the Si network and the formation of CaF_2_ by the reaction of CME with HF, which in effect reduces the Ca available to disrupt the Si network. Importantly, when HF was not used, calcium from CME was incorporated into the hybrid systems better than when using CaCl_2_. Hydroxyapatite (HA) was formed on all class II hybrids in SBF, indicating all the samples were bioactive. The rate of hybrid dissolution was strongly influenced by the type of Ca source used, CME was successful at cross-linking the polymer; hence a steady polymer release was observed whereas in the CaCl_2_ samples a burst release was observed. Class II hybrids synthesised with CME showed compressive strength similar to 70S30C glass stabilised to 700 °C and the presence of Ca in the hybrid improved the modulus of the hybrid two-fold, indicating CME is a promising calcium source for hybrid synthesis.

## Experimental Section

### Sample preparation

The γ-PGA was purchased in the free acid powder form from Natto Biosciences (Quebec, Canada) with 95 wt % polymer and 5 wt % water. All other materials were purchased from Sigma–Aldrich and used as received.

Calcium methoxyethoxide (CME) was prepared following the method in literature.[7b, 21] In brief, calcium granules (1 g) were reacted with 2-methoxyethanol (24 mL) under an argon atmosphere at 80 °C for 24 h. The resultant solution was centrifuged at 4000 rpm for 10 min to remove unreacted calcium metal. The concentration of CME in solution was confirmed gravimetrically by heating to 1050 °C for 12 h, upon which the solvent evaporated and the alkoxide converted to CaO.

Class II calcium-free hybrids and hybrids with either CME or calcium chloride as the calcium source were synthesised in this work. The weight % of organic/inorganic material was 50:50, whereas the molar ratio of Si/Ca was 70:30 to make the inorganic component the same composition as 70S30C (70 mol % SiO_2_, 30 mol % CaO) bioactive glasses. Poly(γ-glutamic acid) (γ-PGA) was used as the organic phase, which was covalently cross-linked to the silica phase (derived from tetraethylorthosilicate (TEOS)) through the organosilane glycidoxypropyltrimethoxy silane (GPTMS). The total organic content of the hybrid was derived from γ-PGA and the organic portion from GPTMS and the inorganic content attributed to the silica derived from TEOS and GPTMS and the CaO from CME.

The synthesis route was modified to that described in previous work.[4p] Here, the use of CME added a level of complexity owing to its high reactivity with water, leading to a three-pot reaction as shown in Scheme 1. First, γ-PGA dissolved in DMSO was reacted with GPTMS at a molar ratio of GPTMS to monomeric glutamic units of 1:2 for 12 h to form the Si-functionalised γ-PGA in solution. The inorganic silica sol was prepared separately by hydrolysing TEOS with H_2_O and HCl (2 N) for 30 min. The Si-functionalised γ-PGA and hydrolysed TEOS were added together and mixed for 5 min, followed by addition of the third pot of CME in methoxy ethanol and DMSO (1:1 v/v). This hybrid precursor mixture was allowed to mix for a further 5 min before casting into Teflon containers for aging (3 days with the containers sealed) and drying (10 days) at 60 °C. Samples were also made with HF to study the influence of HF on gelling and silica network formation. HF (5 wt %) was added to the hybrid precursor sol after mixing pots 1 (Si-functionalised γ-PGA) and 2 (hydrolysed TEOS) at a hybrid sol/HF volume ratio of 17:1. These samples were denoted as 2ECCa_ME_, where 2 corresponds to two moles of glutamic acid (E) to GPTMS (C), with calcium (Ca) from the CME source. Samples made without the addition of HF were denoted as 2ECCa_ME_*.

Class II hybrids made with calcium chloride were synthesised by using the procedure developed by Poologasundarampillai et al.[4p] The γ-PGA was reacted with GPTMS in DMSO for 12 h under an N_2_ atmosphere. This was then rotary-vacuum evaporated to partially remove the DMSO. To this, calcium chloride, which was pre-dissolved in water to aid solvation, was added. After mixing for 30 min, the hydrolysed sol was added and mixed for another 1 h. The hybrid precursor solution was then cast in polymethylpentene (PMP) moulds and HF was added at a volume ratio of hybrid precursor sol to HF of 1:17. These samples, denoted as 2ECCa_Cl_, were aged and dried at 60 °C.

Calcium-free hybrids (denoted as 2EC) were produced following the same procedure as that for the synthesis of 2ECCa_ME_, but no calcium was added. For the synthesis of the 2EC and 2ECCa_ME_ samples, the overall ratio of Si^4+^/H_2_O/H^+^ were 1:2:2, whereas that of 2ECCa_Cl_ was 1:12:2.

### Characterisation

Several analysis techniques were used to study the atomic (MAS NMR), molecular (FTIR and XRD), nano- (SEM) and macro-scale (SIMS) structure and composition of the hybrids. All ^29^Si solid-state magic-angle spinning nuclear magnetic resonance (MAS NMR) data were acquired at 7.05 T on a Varian InfinityPlus 300 spectrometer operating at a ^29^Si Lamor frequency of 59.62 MHz. These measurements were undertaken by using a Varian 7.5 mm probe spinning at 5 kHz. A 5 μs excitation pulse (flip angle ∼π/4) was applied with a 240 s recycle delay to ensure complete relaxation was obtained for each measurement. All ^29^Si MAS NMR data were referenced to the IUPAC recommended primary reference of TMS (*δ*=0 ppm) by a secondary reference of kaolinite located at *δ*=−92 ppm. To quantify the T^*n*^ and Q^*n*^ distribution of the Si speciation, each resonance in the ^29^Si MAS NMR data was simulated with a Gaussian peak shape by using the Origin 8.5 Pro software package (Origin 8.5 Pro, OriginLab, Northampton). X-ray diffraction was performed on a Bruker D2 Phaser, using a step-scanning method with Cu_Kα_ radiation at 30 kV and 10 mA with a count rate of 0.25 s per step, from 2*θ* values of 10° to 70°. Attenuated total reflectance Fourier transform infrared spectroscopy (ATR-FTIR) was used to study the chemical structures of the hybrids. Samples were analysed on a Thermo Scientific Nicolet iS10 FTIR.

### Compositional homogeneity

Secondary ion mass spectroscopy (SIMS) was performed with a TOF-SIMS V instrument (ION-TOF GmbH, Germany) to study the homogeneity of species throughout the hybrids. The samples were polished to a mirror finish to obtain smooth surfaces for SIMS analyses. An area of 500×500 μm^2^ was sputtered with a 10 keV 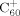 cluster ion source under a current of 1.2 nA for 1 s every scan. After which, an area of 250×250 μm^2^ was analysed by rastering over by a 25 keV 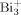 beam with an average current of 0.55 pA. An image of 256×256 pixels for a total of 840 scans was collected. A low-energy electron gun of 20 eV was used for charge compensation.

### Topography

Scanning electron microscopy (SEM) was used to examine the morphological and textural features of chromium-coated hybrids. A Leo 1525 with Gemini column fitted with a field emission gun at 5 kV, working distance between 5–10 mm, with a 30 μm aperture and In-lens secondary electron detector (SEI) was used. All the samples were coated with chromium at 70 mA for 1 min.

### Mechanical properties of the hybrids

Uni-axial compression tests were performed on cylindrical hybrid samples with 5 mm diameter and heights ranging from 2–10 mm. Tests were performed on a Zwick 1474 fitted with a 100 kN load cell at 8.33 mm s^−1^ strain rate until failure or 80 % maximum deformation.

Nanoindentation was performed on the hybrids and inorganic sol-gel glass 70S30C by using a testing procedure similar to that adopted by Mammeri et al.[22] A loading rate of 5 mN s^−1^ to maximum indentation load of 50 mN was applied. A dwell for 20 s at maximum load was applied before unloading at 10 mN s^−1^ to 5 mN, when a final dwell for 60 s was also applied to determine the thermal drift contribution of the indentation system to total displacement measured by a capacitive transducer. A NanoTest Vantage (Micro Materials Ltd, UK) was used to perform the nanoindentations by using a Berkovich pyramidal tip.

### Dissolution in TRIS buffered solution

Hybrid samples were soaked in TRIS buffer to study the dissolution behaviour over time. The release of polymer and dissolution of Si species and Ca^2+^ ions from the hybrids often reveal a large amount of information on the structure and bonding present between the constituents of the hybrid. TRIS buffer was prepared following the procedure outlined in ref. [23], where tris (hydroxymethyl) aminomethane (15.090 g) was dissolved in deionised water (2000 mL), to which HCl (1 n) was added at 37 °C to adjust pH to 7.30. Hybrid monoliths weighing 150 mg were soaked in TRIS (100 mL) and incubated at 37 °C in an orbital shaker set to 120 rpm. After 1, 2, 4, 8, 24, 168 and 336 h of soaking, 1.5 mL solution was aliquoted for Si and Ca elemental analysis and γ-PGA concentration determination. To compensate for the removed 1.5 mL solution, 1.5 mL of fresh TRIS buffer solution was added to the original test solution. The soluble silica species and Ca^2+^ ion concentrations in the aliquoted solutions were analysed by inductive coupled plasma optical emission spectroscopy (ICP-OES, Thermo iCAP 6300 Duo). γ-PGA release into the TRIS buffer solution at each time point was obtained by performing a Pierce Micro bicinchoninic acid (BCA) protein assay. Polymer concentration in solution was determined by reference to a standard curve consisting of known concentrations of functionalised γ-PGA in TRIS buffer solution. The known concentration of standard solutions (1, 50, 100, 200, 300, 500, 750 and 1000 μg mL^−1^) were prepared by first reacting γ-PGA with GPTMS (molar ratio of GPTMS to monomeric glutamic molecule was 1:2) in DMSO. Then, appropriate amounts of the reacted solution were added to TRIS buffer to make up the required standards. An Anthos 2020 spectrophotometer with measurement filter of 562 nm was used to analyse the absorbance of each of the standards and samples.

FTIR, SEM and TGA-DSC were performed on the samples that were incubated in TRIS buffer solution for 672 h to study the effect of dissolution on the hybrid.

### In vitro “bioactivity” testing in SBF

The rate of HCA formation in simulated body fluid (SBF) was determined. Monoliths (150 mg) were immersed in SBF (100 mL) and placed in an orbital shaker with an agitation rate of 120 rpm at 37 °C. 1 mL of each sample was collected at each time point (1, 2, 4, 8, 24, 72, 168, 336 and 672 h) for analysis with ICP-OES and 1 mL of fresh SBF was added to keep the solution volume of 100 mL. A major reduction in phosphorus concentration in SBF was observed after 72 h and 168 h of soaking, therefore to detect HCA layer formation, the samples were filtered (1 μm paper) after 72 and 168 h of soaking and the monoliths were rinsed with acetone, dried and then evaluated by XRD and FTIR. XRD was carried out on ground samples with a PANalytical X′Pert Pro MPD series automated spectrometer, using a step-scanning method with Cu_Kα_ radiation at 40 kV and 40 mA with a 0.040° 2*θ* step and a count rate of 50 s per step, from 2*θ* values of 5° to 75°. Samples for FTIR were ground with potassium bromide (KBr) with a 1:100 weight ratio. The resultant powder was pressed into a pellet and FTIR was performed on a Bruker Vector 22 thermal gravimetric infrared spectrometer (TGA-IR) through transmission with a wavelength of 633 nm and in the range of 400 to 2000 cm^−1^.

### Cell culture

To examine the effect of the new 2ECCa_ME_* hybrids on cell toxicity and growth, hMSCs were seeded directly on the surface of the hybrid monoliths. The hybrid 2ECCa_ME_* monoliths (5×5 mm^2^) were sterilised by immersion in 70 % (v/v) ethanol followed by extensive washing in phosphate buffered saline (PBS) and then incubation in Dulbecco’s modified Eagle′s medium (DMEM), supplemented with penicillin (50 U mL^−1^), streptomycin (50 μg mL^−1^) and amphotericin B (2.5 μg mL^−1^), at 37 °C overnight (preconditioning) prior to cell culture experiments. hMSCs were cultured in low glucose phenol red-free DMEM, supplemented with 10 % fetal bovine serum (FBS), l-glutamine (2 mm), penicillin (50 U mL^−1^), streptomycin (50 μg mL^−1^) and amphotericin B (2.5 μg mL^−1^), all from Invitrogen, UK, (cell culture medium) in a humidified incubator at 37 °C and 5 % CO_2_.

For direct culture on the monoliths, the hMSCs were resuspended in a small volume (20 μL) of cell culture medium, seeded on the surface of the hybrids and incubated for 1 h at 37 °C and 5 % CO_2_. Then, the appropriate amount of medium was added to cover each hybrid (i.e., 0.5 mL/well, 48-well plate). The seeding density used was 1×104 cells cm^−2^ for all experiments.

Cytotoxicity was evaluated with the LIVE/DEAD viability/cytotoxicity assay (Molecular Probes, UK), which was performed according to the manufacturer’s instructions. Stained samples were examined under an Olympus BX-URA2 fluorescence microscope. Images were captured by using a Zeiss Axiocam digital camera and analysed by using KS-300 software (Imaging Associates).

For cell growth, the Alamar Blue assay was used to measure the metabolic activity and consequently the rate of cell growth after 4 and 12 days of culture. As a positive control, hMSCs were seeded on tissue culture plastic and cultured in parallel with the hybrids. For each Alamar Blue measurement, the hybrids were moved to a new plate and incubated at 37 °C and 5 % CO_2_ for 2 h with phenol-free DMEM (without added serum) containing 10 % (v/v) Alamar Blue solution (Invitrogen, UK). Then, 100 μl of each sample was transferred to a new black-with-clear-bottoms 96-well plate and the fluorescent intensities were measured by using a microplate reader, with fluorescence excitation and emission wavelengths of 570 and 610 nm, respectively. To determine the effect of the dissolution products released over time by the hybrids on hMSCs, the metabolic activity of the cells on the plate was measured as well with the Alamar Blue assay. The fluorescence values obtained were averaged, and the average was expressed as a percentage of the cells on cell culture plastic at day 4. Experiments were performed in triplicate, six hybrids for each experiment. The statistical significance of the obtained data was assessed by one-way ANOVA variance analysis by using the Sigma stat software (SigmaStat 4.0, Systat Software, California) and level of significance was set at *p*<0.001.

## Supporting Information

As a service to our authors and readers, this journal provides supporting information supplied by the authors. Such materials are peer reviewed and may be re-organized for online delivery, but are not copy-edited or typeset. Technical support issues arising from supporting information (other than missing files) should be addressed to the authors.

miscellaneous_information
