# Supplementary material for: Poly(γ-glutamic acid)/Silica Hybrids with Calcium Incorporated in the Silica Network by Use of a Calcium Alkoxide Precursor
Source: Chemistry. 2014 May 18;20(26):8149–60. doi: 10.1002/chem.201304013 (PMC4506526; doi:10.1002/chem.201304013)

CHEMISTRY
A European Journal

Supporting Information

© Copyright Wiley-VCH Verlag GmbH & Co. KGaA, 69451 Weinheim, 2014

Poly(γ-glutamic acid)/Silica Hybrids with Calcium Incorporated in the Silica Network by Use of a Calcium Alkoxide Precursor

Gowsihan Poologasundarampillai,*[a] Bobo Yu,[a] Olga Tsigkou,[a, b] Daming Wang,[a] Frederik Romer,[c] Vineet Bhakhri,[a] Finn Giuliani,[a] Molly M. Stevens,[a, b] David S. McPhail,[a] Mark E. Smith,[c, d] John V. Hanna,[c] and Julian R. Jones*[a]

chem_201304013_sm_miscellaneous_information.pdf

Supporting information 1: Mechanical properties of Class II γ-PGA/bioactive silica hybrids synthesised with calcium chloride (CaCl) or calcium methoxyethoxide (CaME) with molar ratios of γ-PGA : GPTMS of 2 dried at 60 °C. Traditional 70S30C bioactive glass synthesised with calcium nitrate and sintered at 800°C was added for comparison. HF was added to the samples 2ECCaCl and 70S30C. (a) Stress/ strain traces (b) Yield stress as a function of height to width (H/W) ratio of the samples.

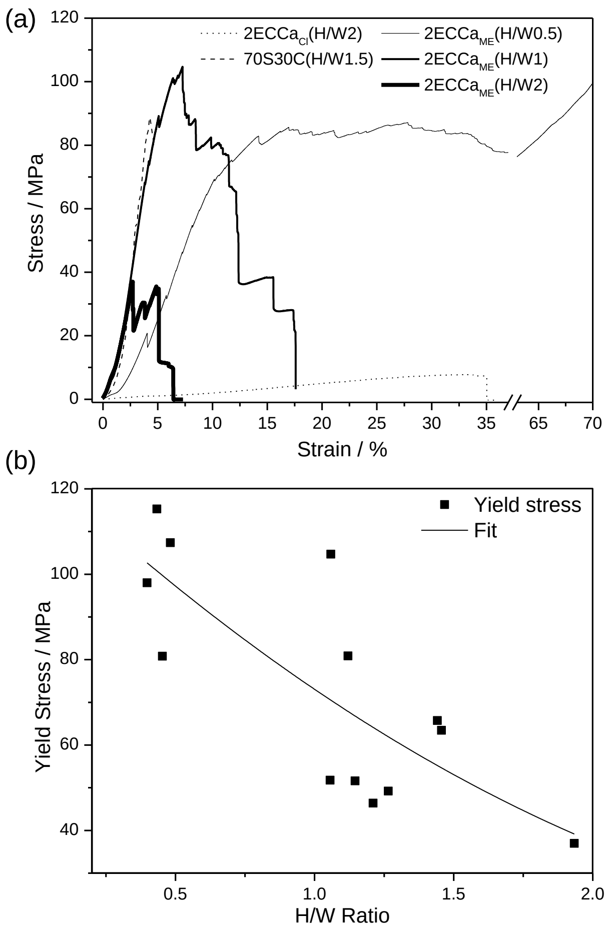

Figure 2. TOF SIMS images (a) Ca+, (b) Si+, (c) SiCaO+, (d) Si2O+, (e) CNOCa2+ and (f) Si2CHO+ of 2ECCaME*: Class II γ-PGA/bioactive silica hybrids synthesised with calcium methoxyethoxide (CaME) with molar ratios of γ-PGA : GPTMS of 2.

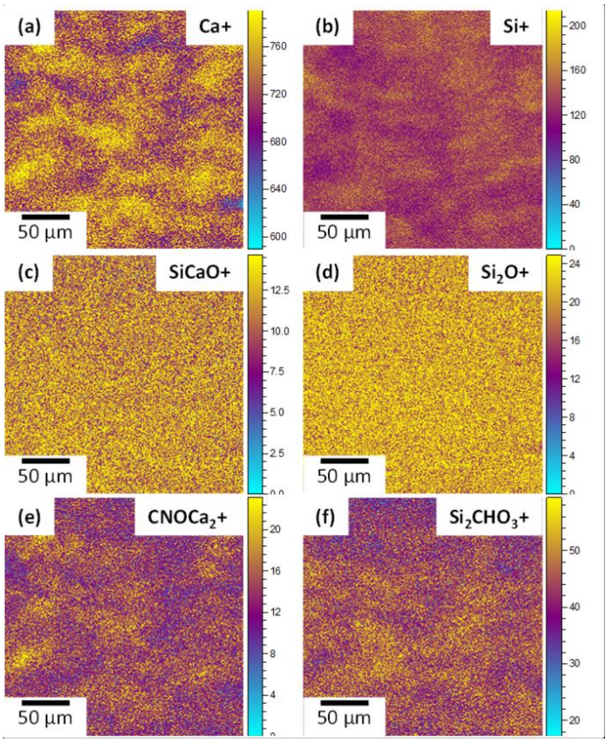

Figure 8. Human bone marrow derived mesenchymal stem cells (hMSCs) viability on 2ECCaME*. (a) LIVE/DEADTM assay of the hMSCs cultured on the hybrids' surface for 7 days (live cells= green, dead cells = red - circled). Cells appear out of focus due to the (b) Metabolic activity as measured with the Alamar Blue assay after 4 and 7 days in culture. * = p<0.001.

(a)

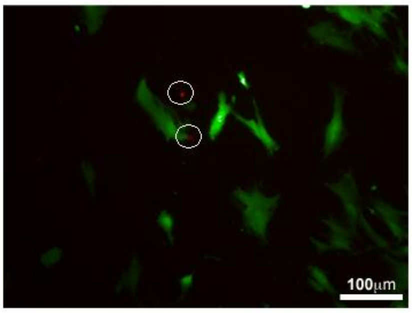

(b)

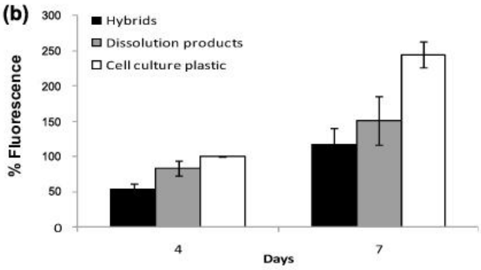

Supplement: Supplementary file 1 — miscellaneous_information [file chem0020-8149-sd1.pdf]
